# MiR-34c Is Predictive of Delayed Cerebral Ischemia After Subarachnoid Hemorrhage

**DOI:** 10.21203/rs.3.rs-6198784/v1

**Published:** 2025-04-01

**Authors:** Bosco Seong Kyu Yang, Sidra Tabassum, Sarah Hinds, Lena M. O’Keefe, Silin Wu, Atzhiry S. Paz, Hua Chen, Aaron M. Gusdon, Xuefang Ren, Huimahn A. Choi

## Abstract

**Introduction** Delayed cerebral ischemia (DCI) is a potentially preventable complication from an aneurysmal subarachnoid hemorrhage (SAH). The micro-RNAs (miR) 34 family has shown its ability to disrupt the blood-brain barrier and redox metabolism and might contribute to the complex pathophysiology of DCI. This study aimsto evaluate the association between the serum levels of miR-34c and the occurrence of DCI. **Methods** This retrospective observational study is based on 72 subjects with acute aneurysmal SAH who were admitted to a single tertiary center between December 2017 and July 2021. Subjects were prospectively adjudicated for clinical outcomes, including delayed cerebral ischemia.Levels of miR-34c were measured in plasma collected within 48 hours of ictus. Patients were median-dichotomized into having a higher or lower plasma level of miR-34c. miR34c levels were compared between DCI and no DCI groups using the Wilcoxon rank sum tests. A multivariable logistic regression model and the Cox proportional hazard model were used to evaluate the effect of higher miR-34c levels. **Results** The median age was 54 years, 76% were females, and 21% developed DCI. Early miR-34c levels were significantly higher in SAH subjects who progressed to have DCI with Cohen’s *d* of 0.75 (p<0.05). Even after adjusting for age, sex, histories of diabetes, hypertension, Hunt-Hess grade, and modified Graeb scores, a higher miR-34c level was associated with 5.7-fold increased odds of DCI (p<0.05; 95% CI: 1.35-32.22). Survival analysis adjusting for the known predictors also revealeda 5.4-fold higher hazard of DCI for the patients with a higher miR-34c level (p < 0.05; 95% CI 1.22-25.43). **Conclusion** The present study demonstrates the potential importance of circulating miR-34c in predicting DCI in SAH patients. Given the known importance of the miR-34 family in vascular physiology, it may be an important target for future studies.

## Introduction

Delayed cerebral ischemia (DCI) is a complication after an aneurysmal subarachnoid hemorrhage (SAH), which occurs 4-21 days after ictus in approximately 20-30% of SAH patients[1]. It is defined as a neurologic deficit or impaired consciousness that lasts more than an hour or as a new ischemic lesion on imaging studies and is strongly associated with poor outcomes[2,3]. Pathophysiologic processes, including vasospasm, microcirculatory dysfunction, cerebral vascular dysregulation, cortical spreading depolarization, microthrombosis, and neuroinflammation, have all been implicated in the pathophysiology of DCI[1,4–6].

Recent studies report that, besides the well-known environmental and vascular risk factors, epigenetic factors also contribute to the risk of ischemic and hemorrhagic stroke[7,8]. MicroRNAs (miRNA, miR) are small RNA molecules coded in non-coding regions that are separately regulated from protein-coding genes, each regulating translational rates of distinct sets of messenger RNAs. miRNA's pleiotropic potential to affect multiple pathophysiologic processes has instigated multiple studies to explore the potential of miRNA as a biomarker or therapeutic agent in all kinds of diseases, including cancer, genetic diseases, neurodegenerative diseases, and SAH[9,10]. Insufficient knowledge of each miRNA's targets and lack of targeted delivery systems and pharmacokinetic/dynamic understanding have deterred its translation into clinical practice, necessitating a better understanding of the role of miRNAs in the development of SAH and its complications.

MiR-34c is a member of the structurally related miR-34 family, composed of three pro-apoptotic members: miR-34a, miR-34b, and miR-34c, all of which have been described as transcriptional targets of p53[11]. Their major roles in oncogenesis converge on the inhibition of tumor growth and metastasis[12]. Potential mechanisms of action of miR-34a and miR-34b in complications of stroke have been described[13,14]. MiR-34a is shown to aggravate injury from ischemic stroke by increasing the blood-brain barrier (BBB) permeability and causing mitochondrial dysfunction[14]. In contrast, miR-34b overexpression has been shown to ameliorate reperfusion injury from revascularized ischemic stroke by counteracting oxidative stress in animal models[15]. The miRNA family's involvement with the homeostasis of BBB, mitochondrial function, and redox metabolism strongly suggests their potential participation in the pathogenesis of DCI. Yet, there is a paucity of focused studies examining miR-34c and cerebrovascular diseases, and no studies examining miR-34c and SAH complications. Therefore, the present study aims to evaluate the association between miR-34c and DCI after SAH.

## Methods

*Study population*

This retrospective observational study is based on SAH subjects admitted to the neuroscience intensive care unit between December 2017 and July 2021 at a single tertiary academic center. Inclusion criteria were adults of age 18 years or older and a severe aneurysmal SAH of modified Fisher scale of 3 or 4, diagnosed by either computed tomography (CT), CT Angiography, or digital subtraction angiography within 24 hours of ictus, the consent to blood sampling, and the availability of blood samples. Exclusion criteria were SAH due to trauma, arteriovenous malformation, mycotic aneurysms, and comorbid conditions that might critically influence the expression of miR-34c, including autoimmune disease and history of malignancy. Medical records, including demographic factors, comorbid conditions, and image studies, were collected during each subject's hospital stay. No previous studies have studied miR-34c levels in SAH patients.

*Informed consent and ethics approval*

The study was conducted with the approval of the institutional review board (IRB: HSC-MS-17-0776). Written informed consent was obtained from the patient or surrogate.

*Classification of DCI and ancillary outcomes*

DCI was defined as the occurrence of focal neurological impairment, such as hemiparesis, aphasia, apraxia, hemianopia, and neglect, or a decrease of at least two points on the Glasgow Coma Scale—either on the total score or on one of its individual components. To qualify as DCI, symptoms needed to last for at least one hour, should not be apparent immediately after aneurysm occlusion, and could not be attributed to other causes through clinical assessment, a CT, or a magnetic resonance imaging (MRI) scan of the brain, and appropriate laboratory studies[16].

DCI was adjudicated through consensus of at least two attending neurointensivists in weekly research meetings. In rare instances when no consensus could be achieved, the principal investigator made the final determination.

*Measurements of circulatory miRNAs*

Blood samples were collected in EDTA-treated tubes within 48 hours of aneurysmal rupture and before the interventions to secure ruptured aneurysms and processed within one hour of collection. The tubes were inverted gently to ensure that they were mixed properly. The tubes were then centrifuged at 1460g for 10 minutes at 4°C to separate plasma from the cellular components. Following centrifugation, the upper plasma layer was carefully transferred to a new sterile 15 ml tube, avoiding disturbance of the buffy coat. Then, the 15mL conical tube was centrifuged at 3260g for 10 minutes at 4 °C. The plasma was removed and aliquoted at 500ul per tube. Storage tubes were then frozen at -80°C for long-term storage. In measuring miRNA levels in the samples, a next-generation sequencing technique-based miRNA quantification assay—the HTG EdgeSeq miRNA transcriptome assay (HTG Molecular Diagnostics, Tuscon, AZ)—was used to detect miRNAs with low expression levels accurately. The preprocessed samples were incubated with the probes targeting a selected set of miRNAs including miR-34c (miR34a-5p, miR34a-3p, miR34c, miR181a, miR15a, miR9a, miRlet7a, miR124, miR6715p, miR4306, miR335, miR1925p, miR1923p, miR5585, miR506, miR520d, miR524, miR5011) for 20 hours, and after additional quality-control steps, sequencing was done with the Illumina NextSeq 500 sequencer (Illumina, San Diego, CA) following the manufacturer's protocol. The generated sequences were processed with Illumina BaseSpace bcl2fastq software version 2.2.0 and HTG EdgeSeq Parser software version v5.3.0.7184 to extract raw counts for each miRNA. The assay included both positive and negative control miRNA probes and read counts for housekeeping genes, including ATBC, B2M, GAPDH, YWHAZ, RPL19, RPS20, RPL27, and RSP12, that ensured accurate quantification. The read count for each miRNA was converted into counts per million (CPM) by dividing each read count by the sum of all the counts in each sample and multiplying by one million. The resulting CPM was log-transformed with a base of two to stabilize variance and then dichotomized to a higher or lower level with the median as a threshold if deemed appropriate to improve interpretability.

*Statistical analysis*

For descriptive analysis, Wilcoxon rank sum tests were used for continuous variables, while Pearson's Chi-squared tests and Fisher's exact tests were used for categorical variables as appropriate. Cohen's d was calculated for the difference in miR-34c levels between SAH patients with and without DCI to determine the effect size. The initial analysis focused on identifying factors that might mediate the relationship between miR-34c and DCI by investigating the associations between patient factors and a miR-34c level. Then, two different aspects of the association between miR-34c and DCI were studied. A multivariable logistic regression model was used to investigate the effect of miR-34c on the odds of DCI, adjusting for patient factors that included age, sex, history of hypertension, diabetes mellitus, modified Graeb score, and the Hunt-Hess scale (HH).  Survival analysis was conducted to examine the temporal homogeneity of miR-34c's effect on the probability of DCI. Kaplan-Meier models with a log-rank test and the Cox proportional hazard model, adjusting for the same set of patient factors, were used. Adjustment for the patient-level factors intended to remove factors that might confound the association between miR-34c and DCI[17–21]. Subjects with missing values for the variables used in the models were excluded. A p-value less than 0.05 was chosen as the threshold for statistical significance.

## Results

*Baseline characteristics of the participants*

A total of 72 SAH subjects were enrolled. The median age was 54 (IQR: 43-62), and 76% were females. Among the SAH subjects, DCI was observed in 21%, consistent with previous reports[22]. The demographics were not different between SAH subjects with DCI and without DCI (Table 1). Furthermore, the prevalence of comorbidities, the severity of SAH measured with HH grades, modified Graeb scores, and modified Fisher scale, and treatment modalities were not different between the two groups ($p > 0.05$).

*Potential associations between patient factors and miR-34c levels*

Patient-related factors, including patients' age, sex, comorbid conditions, HH grades, the modified Graeb scores, the need for mechanical ventilation, and the modality of interventions, did not show significant associations with miR-34c levels (*Table 2*).

*Effects of miR-34c expression levels onthe odds of DCI*

The influence of miR-34c levels on the subjects' odds of having DCI were investigated. Without adjustment, SAH subjects with and without DCI showed significantly different distributions of miR-34c levels ($p < 0.05$) (*Figure 1*). Based on an unadjusted logistic regression model, having a higher miR-34c level increased the odds of DCI by 3.52 folds ($p<0.05$; 95% CI: 1.06-13.93) compared to the subjects with lower miR-34c levels. When the model was adjusted for age, sex, histories of diabetes mellitus, hypertension, the HH grades, and the modified Graeb score, a higher miR-34c level showed a statistically significant increase in the odds of DCI with the adjusted odds ratio of 5.75 ($p<0.05$; 95% CI: 1.35-32.22) (*Figure 2*).

*Temporal homogeneity of the effects of miR-34c expression levels*

The median onset of DCI was 7 days [IQR: 6-11 days] after aneurysmal rupture. Based on the Kaplan-Meier model, subjects with higher and lower miR-34c levels did not show significant differences ($p > 0.05$) (*Figure 3*). However, the Cox proportional hazard model adjusting for age, sex, histories of diabetes mellitus, hypertension, modified Graeb score, and the HH grades revealed that the subjects with higher miR-34c levels had a 5.44-fold higher hazard of DCI than the subjects with lower miR-34c levels ($p < 0.05$; 95% CI 1.21-24.50; *Table 3*). The Schoenfeld residual test did not show a violation of proportional hazard assumptions.

## Discussion

Our main finding is that higher plasma levels of miR-34c in the first 48 hours are significantly associated with DCI. miR-34c expression significantly differed between SAH subjects with and without DCI. Among SAH subjects, the odds of having DCI were significantly higher for those with higher levels of miR-34c, even after adjusting for patient factors, including age, sex, histories of diabetes mellitus, hypertension, modified Graeb score, and HH grades. Survival analysis revealed that subjects with higher miR-34c levels showed a higher probability of developing DCI when adjusted for patient factors using the Cox proportional hazard model.

*miRNA as a biomarker for outcomes and complications from SAH*

Levels of circulating miRNAs undergo unique alterations in various disease conditions such as cancer, diabetes mellitus, hypertension, myocardial infarction, and heart failure[23]. In SAH, previous studies examining circulating miRNAs have shown that the prognostic performance of prediction models is improved by including selected miRNAs in models[10,24]. For instance, mutations that downregulate the functions of miR-155 have been linked to increased incidence of aneurysmal rupture[25]. An increased plasma level of miR-502-5p has shown associations with poor outcomes at one year in SAH patients[26].

Our finding is particularly notable because of its novelty and the strong association between miR-34c and DCI. The association's magnitude and persistence, despite adjustment for patient factors, make miR-34c a promising biomarker for DCI. One previous study analyzed 754 miRNAs, including miR-34c, in the cerebrospinal fluid (CSF) samples in SAH patients with and without DCI and found none of the miRNAs to be differentially expressed between the two populations[27]. Multiple reasons may account for the difference. Our analytic technique allowed us to skip the RNA extraction step during miRNA analysis, which might have lowered the possibility of erroneous loss of samples and the introduction of extraction-related bias. Also, our study involved a larger number of patients, and the difference in the analyzed compartment—plasma and CSF—might have caused the difference. Stark differences in the expression profiles for the same miRNA between the two compartments and generally larger magnitude of changes in the miRNA expression in the plasma compartment have been observed, further emphasizing the importance of our finding[28].

The temporal gap between when the plasma sample was collected and when DCI developed accentuates the importance of our finding. Not only is the admission level of miR-34c associated with increased overall odds of later complications, but its influence on the probability of DCI persisted over time, as evidenced by the Cox proportional hazard model. This temporal gap marks an important difference that our study holds in comparison to previous studies, which found miRNA biomarkers of DCI in the samples collected at 5 and 7 days post-ictus[27,28]. This finding suggests that miR-34c might be a marker of the deterministic pathophysiological mechanisms during the early phase of SAH that eventually might lead to DCI. The current consensus on the mechanisms of brain injury resulting from SAH goes along with this concept. The futility of treating and preventing delayed complications in improving eventual functional outcomes has shifted our focus to the ultra-early complex combination of pathologic molecular, mechanical, and cellular processes following SAH[29,30]. An increasing number of studies suggest that a pathologic process that occurs within 72 hours of ictus—early brain injury (EBI)—might be the most important predictor of outcome. In addition, numerous preclinical studies targeting EBI have shown significant functional improvements in animal models, further highlighting the importance of EBI[31].

*Pathophysiological roles played by miRNA*

Identifying the exact pathophysiological roles played by miRNAs is key to facilitating miRNA's translation into clinical practice. Their crucial roles in the pathogenesis of various neurodegenerative and cardiovascular diseases are already proven[32–34]. Complications from SAH have not benefited from such detailed mechanistic studies. The only study that analyzed miRNA's role in the pathogenesis of DCI has identified miR-4463, miR-4532, miR-4793, and miR-1290 as the key players and suggested their disruption of neurogenesis as the pathophysiological link. However, only a few experiments support those miRNAs' actual existence, and their reported sequences lack consensus, further questioning the genuineness of the finding[35–40].

The miR-34 family is a well-described group of miRNAs involved in regulating apoptosis[11]. Their existence has been corroborated by numerous experiments, and they show higher abundance and strong sequence consensus[36–40]. In clinical applications, the miR-34 family has been targeted in cancer studies[41]. Specifically, the up-regulation of miR-34c has been shown to induce cell apoptosis[42]. In neurological disorders, miR-34c has been implicated in the pathogenesis of Alzheimer's Disease and cognitive decline in major depressive disorders[43,44]. A study demonstrated that miR-34c facilitates neuroinflammation in drug-resistant epilepsy, suggesting an important role of miR-34c in neuroinflammation. Recently, an in-vitro study of ischemic/reperfusion injury and an in-vivo study of spinal cord injury demonstrated a critical role of miR-34c in the pathophysiology of the disease via an inflammatory and apoptotic pathway[45–47]. Inflammatory mechanisms have been implicated in the pathophysiology of DCI and poor functional outcomes after SAH[48]. Studies also demonstrate the potential role of miR-34c in modulating vascular smooth muscle cell proliferation by either targeting stem cell factor (SCF) or high mobility group box protein-1 (HGMB1)- a pro-inflammatory mediator[49,50]. Notably, cerebral vasospasm is a common complication of SAH and is highly associated with DCI[51,52]. This evidence suggests the underlying pathophysiological mechanisms explaining the association between miR-34c and DCI.

Our study focused on plasma miRNA profiles associated with DCI. However, studies examining both central and systemic roles of miRNAs are needed. Elevated levels of the miRNAs let-7b-5p, miR-19b-3p, miR-125-5p, miR-221-3p, miR-21-5p, and miR-27a-3p in the CSF have been linked to a higher likelihood of delayed cerebral vasospasm in patients with aneurysmal SAH[24]. However, in the plasma, a different set of miRNAs, let-7a-5p, miR-146a-5p, miR-204-5p, miR-221-3p, miR-23a-3p, and miR-497-5p, showed correlations with delayed cerebral vasospasm[53]. The observed difference between miRNA profiles in the CSF and plasma agrees with the current understanding of DCI, which involves a complex interplay between the two compartments[54].

*Limitations*

There are significant limitations to this study. First, it is based on a single center, which limits generalizability and necessitates further validation in different cohorts. The study was based on convenient samples, and its small sample size further limits its generalizability and necessitates further validation.  Second, the discovered associations are not evidence of causation, meaning it is impossible to define the exact role played by miR-34c. Whether miR-34c is protective, detrimental, or a physiologic bystander in the pathophysiological process of brain injuries from SAH leading to DCI cannot be determined by our study alone. Third, our analysis was agnostic to the specific cellular subcompartment where miR-34c might be active. MiRNA exists in the nucleus, cytoplasm, and extracellularly. We analyzed circulating miR-34c levels, which incorporate miRNAs found in plasma and extracellular vesicles. Further studies are required to investigate whether miR-34c's association with DCI stems from its local or distant translational regulation. Fourth, we only focused on patients with severe SAH to ensure that the study population includes a sufficient proportion of patients with DCI and isolate the association of miR-34c and DCI from the severity of SAH—a potentially strong confounder—which restricts the study's generalizability. Furthermore, in addition to the complexity of SAH, its treatments involve a multitude of medications and interventions, and the possibility that miR-34c is a response to the treatments instituted in the hospital cannot be excluded. Finally, due to the inherent limitations of DCI as an SAH outcome, the clinical importance of miR-34c needs to be validated with further studies using functional outcomes, such as the modified Rankin scale and the Glasgow outcome scale, as dependent variables[55]. Future translational studies examining the clinical significance of miR-34c in the pathophysiology of DCI are needed.

## Conclusion

DCI is a delayed complication of SAH that is correlated with poor outcomes. Our lack of understanding of underlying pathophysiological mechanisms deprives us of effective diagnostic and interventional strategies. Our findings present evidence of a strong correlation between the plasma level of miR-34c and the odds of DCI. Survival analysis supported this finding by showing the temporal consistency of early miR-34c levels and their effect on the risk of DCI. Further studies are needed to investigate the potential mechanism connecting miR-34c to DCI.

## Abbreviations

SAH- subarachnoid hemorrhage; DCI- delayed cerebral ischemia; miRNAs- microRNAs, CT- computed tomography, mRS- modified Rankin score, SCF- stem cell factor, HGMB1- high mobility group box protein-1.

## Declarations

The authors confirm that the manuscript complies with all instructions to the authors.

All authors read and approved the final manuscript before submission.

This article has not been submitted or published elsewhere.

The STROBE checklist was used to ensure that the current study meets reporting standards (von Elm, E., Altman, D. G., Egger, M., Pocock, S. J., Gøtzsche, P. C., Vandenbroucke, J. P., & STROBE Initiative (2007). The Strengthening the Reporting of Observational Studies in Epidemiology (STROBE) statement: guidelines for reporting observational studies. Annals of internal medicine, 147(8), 573–577. https://doi.org/10.7326/0003-4819-147-8-200710160-00010)

**Acknowledgments**:

None

**Disclosure** :

HAC has received consultant fees from Grace pharmaceuticals.

**Conflicts of Interest**:

HAC is an editor of the journal, *Translational Stroke Research.*

**Funding: HAC received support from the NIH under award 1R61NS119640-01A1. XR received support from the National Institutes of Health (1R01NS117606-01A1), National Science Foundation (1916894), and new faculty start-up funds from the University of Texas Health Science Center at Houston. AMG received support from National Institute of Neurological Disorders and Stroke under award K23NS121628.**

**Informed consent and ethics approval:** The study was conducted with the approval of the institutional review board (IRB number HSC-MS-17–0776, HSC-MS-12–0637 and HSC-MH-17–0452). Written informed consent was obtained from the patient or surrogate.

**Data Availability:** The data that support the findings of this study are available from the corresponding author upon reasonable request.

**Author contributions**:

Bosco Seong Kyu Yang, Sidra Tabassum, Huimahn Alex Choi: conceptualization, methodology, formal analysis, investigation, writing – original draft, writing – review & editing, visualization

Sarah Hinds, Lena O'Keefe, Silin Wu, Athziry Paz, Hua Chen: methodology, validation, formal analysis, data curation, visualization

Aaron Gusdon, Xuefang Ren: validation, writing – review & editing, supervision

## Tables

**Table 1. Baseline patient characteristics.**

| Characteristic | **DCI (-)**, N = 57[1] | **DCI (+)**, N = 15[1] | **p-value**[2] |
|---|---|---|---|
| Age (Median, IQR) | 53 (42, 63) | 54 (47, 59) | 0.9 |
| Sex | | | 0.5 |
| Female | 42 (74%) | 13 (87%) | |
| Male | 15 (26%) | 2 (13%) | |
| Diabetes mellitus | 8 (17%) | 2 (14%) | >0.9 |
| | | | |
| Hypertension | 38 (83%) | 10 (71%) | 0.5 |
| | | | |
| Hunt-Hess | | | 0.2 |
| Good ( < 3) | 14 (25%) | 1 (6.7%) | |
| Poor | 43 (75%) | 14 (93%) | |
| Modified Fisher Scale | | | 0.3 |
| 3 | 45 (79%) | 10 (67%) | |
| 4 | 12 (21%) | 5 (33%) | |
| Modified Graeb score | 5 (0, 10) | 5 (1, 11) | 0.5 |
| Mechanical Ventilation | 33 (58%) | 12 (80%) | 0.12 |
| Surgical intervention | | | 0.4 |
| Clipped | 20 (35%) | 3 (20%) | |
| Coiled | 37 (65%) | 12 (80%) | |
| miR-34c | 17.4 (5.9, 19.4) | 19.4 (18.0,21.3) | _**0.007***_ |

[1]Median, (Interquartile range); n (%)

[2]Wilcoxon rank sum test; Fisher's exact test; Pearson's Chi-squared test

*p-value < 0.05

**Table 2. Associations between Patient Factors and miR-34c levels**

| Variables | | Odds Ratios (estimate, 95% CI) | p-value |
|---|---|---|---|
| **Age** | | 0.99 (0.95 to 1.03) | 0.58 |
| **Sex** | Female | *Ref.* | |
| | Male | 1.59 (0.53 to 4.97) | 0.41 |
| **Diabetes Mellitus** | No | *Ref.* | |
| | Yes | 0.78 (0.18 to 3.08) | 0.73 |
| **Hypertension** | No | *Ref.* | |
| | Yes | 3.00 (0.93 to 14.78) | 0.13 |
| **Hunt-Hess** | Good ( < 3 ) | *Ref.* | |
| | Poor | 1.67 (0.53 to 5.56) | 0.39 |
| **Modified Graeb score** | | 1.07 (0.98 to 1.16) | 0.13 |
| **Intubation** | No | *Ref.* | |
| | Yes | 1.43 (0.55 to 3.78) | 0.47 |
| **Surgical Intervention** | Clipped | *Ref.* | |
| | Coiled | 1.47 (0.54 to 4.06) | 0.45 |

**Table 3. Adjusted Hazard Ratios of DCI in SAH Patients**

| Variable | Adjusted Hazard Ratios (95% CI) | p-value |
|---|---|---|
| **miR-34c** | | |
| Lower | *Ref.* | - |
| Higher | 5.44 (1.21, 24.50) | ***0.03**** |
| **Age** | 1.01 (0.95, 1.06) | 0.85 |
| **Sex** | | |
| Female | *Ref.* | - |
| Male | 0.78 (0.15, 4.08) | 0.77 |
| **Diabetes mellitus** | | |
| No | *Ref.* | - |
| Yes | 0.82 (0.16, 4.11) | 0.81 |
| **Hypertension** | | |
| No | *Ref.* | - |
| Yes | 0.25 (0.06, 1.07) | 0.06 |
| **Modified Graeb Score** | 0.99 (0.89-1.10) | 0.84 |
| **Hunt-Hess** | | |
| Good ( < 3 ) | *Ref.* | - |
| Poor | 2.89 (0.34, 24.44) | 0.33 |

*p-value < 0.05

## Figures

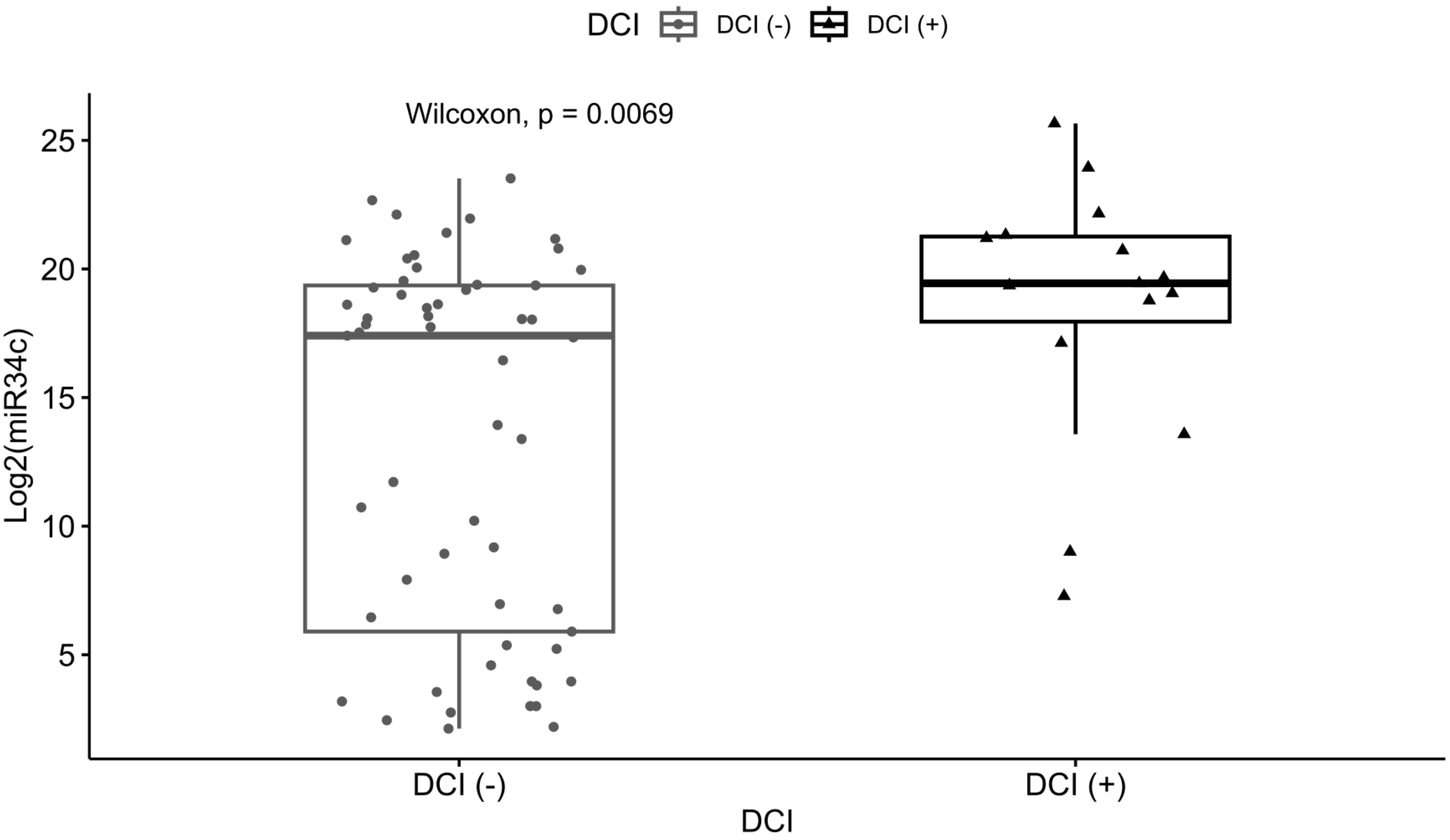

### Figure 1

#### Correlations between Outcomes and miR-34c levels

MiR-34c levels within 48 hours of ictus are statistically significantly different in subjects who proceed to develop delayed cerebral ischemia (DCI).

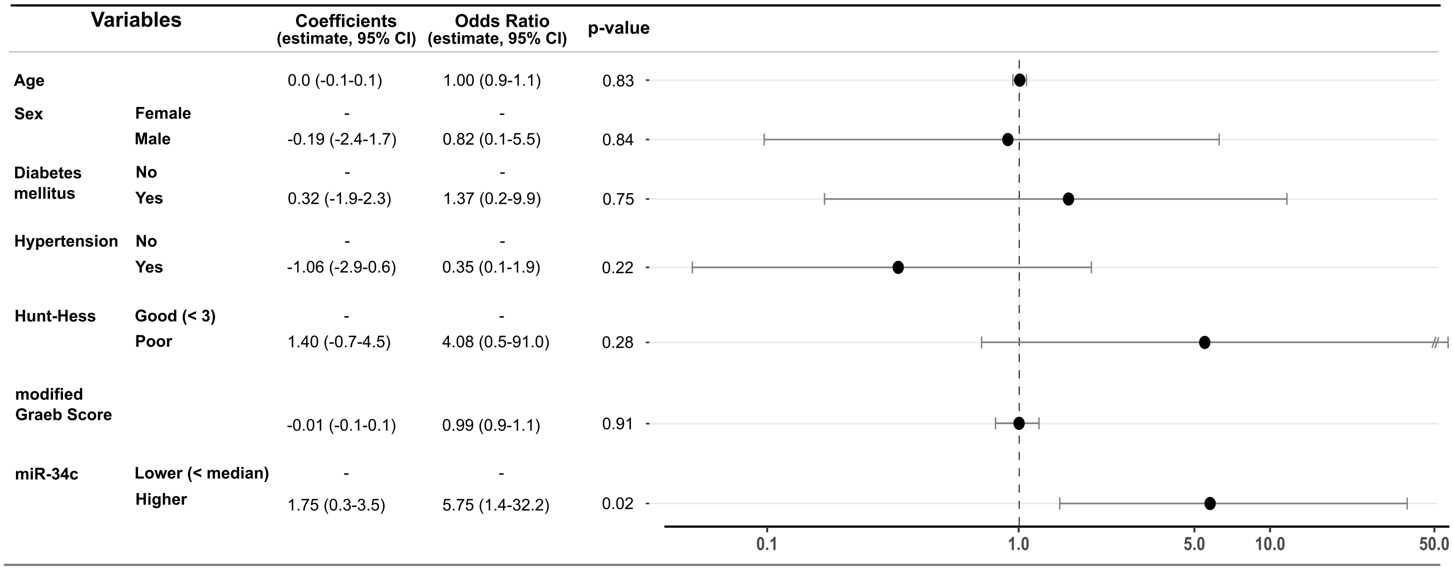

| Variables | | Coefficients (estimate, 95% CI) | Odds Ratio (estimate, 95% CI) | p-value |
|---|---|---|---|---|
| Age | | 0.0 (-0.1-0.1) | 1.00 (0.9-1.1) | 0.83 |
| Sex | Female | - | - | |
| | Male | -0.19 (-2.4-1.7) | 0.82 (0.1-5.5) | 0.84 |
| Diabetes mellitus | No | - | - | |
| | Yes | 0.32 (-1.9-2.3) | 1.37 (0.2-9.9) | 0.75 |
| Hypertension | No | - | - | |
| | Yes | -1.06 (-2.9-0.6) | 0.35 (0.1-1.9) | 0.22 |
| Hunt-Hess | Good (< 3) | - | - | |
| | Poor | 1.40 (-0.7-4.5) | 4.08 (0.5-91.0) | 0.28 |
| modified Graeb Score | | -0.01 (-0.1-0.1) | 0.99 (0.9-1.1) | 0.91 |
| miR-34c | Lower (< median) | - | - | |
| | Higher | 1.75 (0.3-3.5) | 5.75 (1.4-32.2) | 0.02 |

### Figure 2

#### Adjusted Odds Ratios of DCI in SAH Patients

Adjusting for patient factors, patients with higher than the median levels of miR-34c were associated with 5.71-times increased odds of DCI [95% confidence interval (CI): 1.35 – 32.22] than those with lower miR-34c levels.

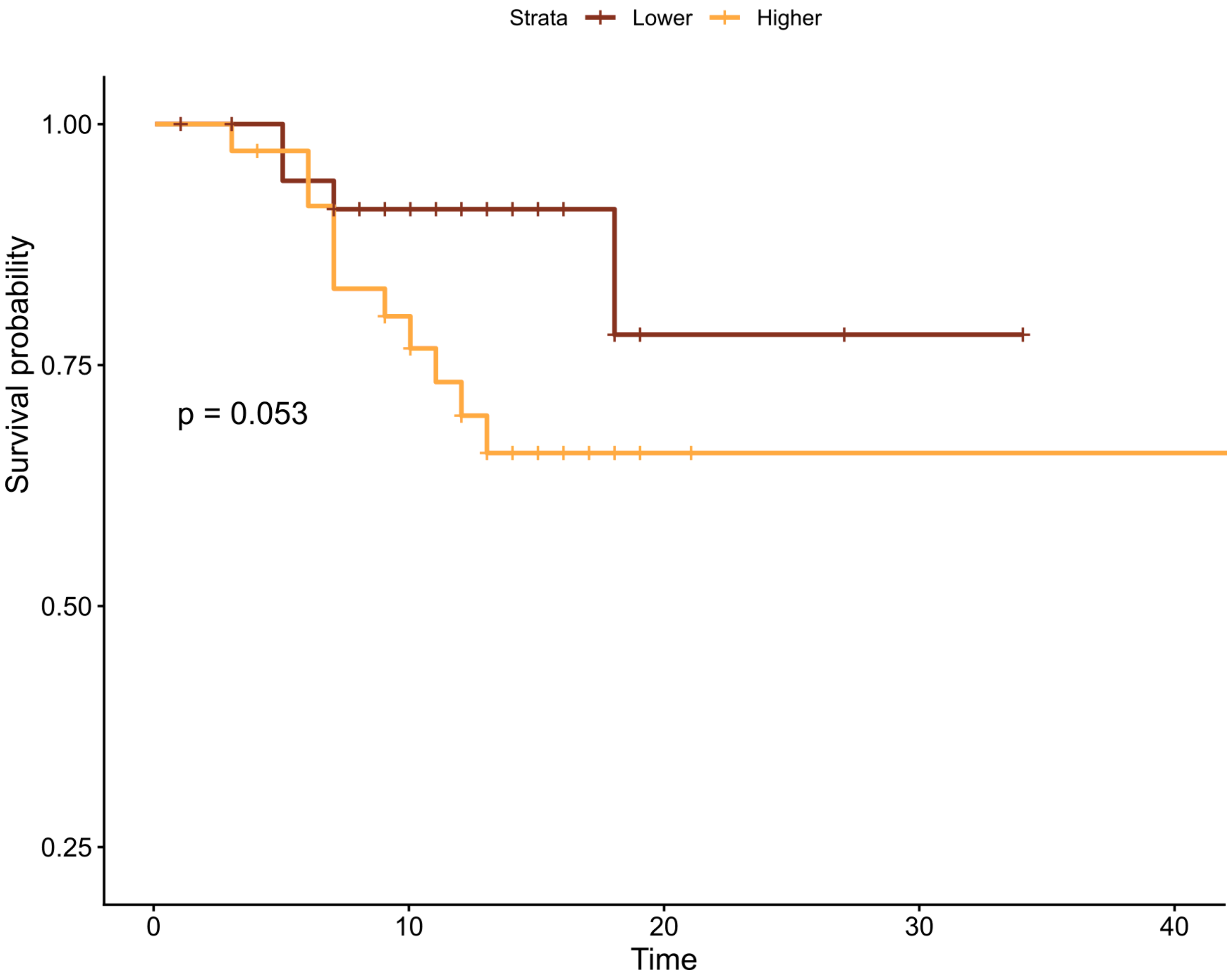

**Figure 3**

**Kaplan Meier survival curves based on differing miR-34c levels**

Patients with higher and lower levels of miR-34c did not show a difference between their probabilities of delayed cerebral ischemia (p > 0.05).

### Supplementary Files

This is a list of supplementary files associated with this preprint. Click to download.

- ht3strobechecklist20250303v1.docx
